# The Regenerative Potential of Amniotic Fluid Stem Cell Extracellular Vesicles: Lessons Learned by Comparing Different Isolation Techniques

**DOI:** 10.1038/s41598-018-38320-w

**Published:** 2019-02-12

**Authors:** Lina Antounians, Areti Tzanetakis, Ornella Pellerito, Vincenzo D. Catania, Adrienne Sulistyo, Louise Montalva, Mark J. McVey, Augusto Zani

**Affiliations:** 1 Developmental and Stem Cell Biology Program, Peter Gilgan Centre for Research and Learning, The Hospital for Sick Children, Toronto, M5G 0A4 Canada; 2 Division of General and Thoracic Surgery, The Hospital for Sick Children, Department of Surgery, The University of Toronto, Toronto, M5G 1X8 Canada; 3 Department of Anesthesia and Pain Medicine, The Hospital for Sick Children, Department of Anesthesia, University of Toronto, Toronto, M5G 1X8 Canada

## Abstract

Extracellular vesicles (EVs) derived from amniotic fluid stem cells (AFSCs) mediate anti-apoptotic, pro-angiogenic, and immune-modulatory effects in multiple disease models, such as skeletal muscle atrophy and Alport syndrome. A source of potential variability in EV biological functions is how EV are isolated from parent cells. Currently, a comparative study of different EV isolation strategies using conditioned medium from AFSCs is lacking. Herein, we examined different isolation strategies for AFSC-EVs, using common techniques based on differential sedimentation (ultracentrifugation), solubility (ExoQuick, Total Exosome Isolation Reagent, Exo-PREP), or size-exclusion chromatography (qEV). All techniques isolated AFSC-EVs with typical EV morphology and protein markers. In contrast, AFSC-EV size, protein content, and yield varied depending on the method of isolation. When equal volumes of the different AFSC-EV preparations were used as treatment in a model of lung epithelial injury, we observed a significant variation in how AFSC-EVs were able to protect against cell death. AFSC-EV enhancement of cell survival appeared to be dose dependent, and largely uninfluenced by variation in EV-size distributions, relative EV-purity, or their total protein content. The variation in EV-mediated cell survival obtained with different isolation strategies emphasizes the importance of testing alternative isolation techniques in order to maximize EV regenerative capacity.

## Introduction

Amniotic fluid stem cells (AFSCs) are a population of broadly multipotent cells that have opened new avenues for regenerative medicine^1^. AFSCs can be isolated via selection of the stem cell factor receptor c-kit (CD117) from human and rodent amniotic fluid, they exhibit clonogenic capability without forming teratomas up to 250 population doublings, and are able to differentiate into all three germ-cell layers^2,3^. Increasingly, AFSCs have been studied in the context of organ and tissue regeneration, including the kidney^4–6^, heart^7^, intestine^8^, lung^9,10^, bone^11^, bladder^12^, and muscle^13,14^. As for their mechanism of action, AFSCs confer beneficial effects in terms of organ regeneration despite a low engraftment rate, thus suggesting a paracrine effect^8–10^. Paracrine intercellular communication by AFSCs and other stem cells relevant to organ regeneration, appear to, at least in part, be mediated by extracellular vesicles (EVs)^15–18^. EVs are small, sub-cellular, biological membrane bound nanoparticles that contain specific cargo in the form of coding and non-coding genetic material, bioactive proteins, and lipids^19–21^. Despite an increasing number of publications studying the role of AFSC-EVs in tissue regeneration, there remain no comparative studies on the isolation of AFSC-EVs^6,22–26^. Since EV regenerative capacities may differ as a result of different isolation strategies, identifying the optimal EV isolation technique is necessary. To examine the effects of different isolation strategies, we collected, isolated, and analyzed AFSC-EVs (adhering to the 2014 recommendations of the International Society for Extracellular Vesicles^27,28^), using isolation techniques based on differential sedimentation (ultracentrifugation (UC)), solubility (ExoQuick, Total Exosome Isolation Reagent (TEIR), Exo-PREP) or size-exclusion chromatography (qEV) (Table 1). We compared these different EV isolation techniques and investigated the impact that each had on the therapeutic potential that AFSC-EVs exert on damaged lung epithelium, as an example of their possible use in regenerative medicine.

**Table 1:** Comparison of the Amniotic Fluid Stem Cell-Extracellular Vesicles (AFSC-EVs) isolation techniques employed in the present study.

Technique	Manufacturer suggested amount	Method	Advantages	Disadvantages
UC	Regulated by centrifuge tube capacity	Pellet EVs at 100,000 g, after pre-clearing CM from cellular debris and live cells	High scalability (up to 32 mL when using Beckman Coulter rotor)	Inconsistent reproducibility across studies due to rotor size, UC time, speed and temperature Impurity of EV pellet due to aggregation of other particles Protocol may take >12 hours
ExoQuick	20% of CM	Reagent based methods that force precipitation of EVs out of solution due to water sequestration	Little processing time, but may require overnight incubation Expensive equipment not needed	Cost per preparation Retention of polymers in reagent
TEIR	50% of CM			
Exo-PREP	100% of CM			
qEV	500 µL of CM at a time, up to 4-time use	Sepharose beads in columns that fractionate CM based on gravity.	High yield of small size EV Low protein contamination Low sheer stress on EVs Fast protocol (15 minutes/preparation)	Column clogs and requires rinsing with NaOH/PBS to ensure adequate flow-through rate

UC: ultracentrifugation.

TEIR: Total Exosome Isolation Reagent.

CM: conditioned medium.

PBS: phosphate buffered saline.

## Results

### Different isolation techniques led to collection of EVs with similar morphology, but different sizes and concentrations

#### Morphology

**Figure 1:**
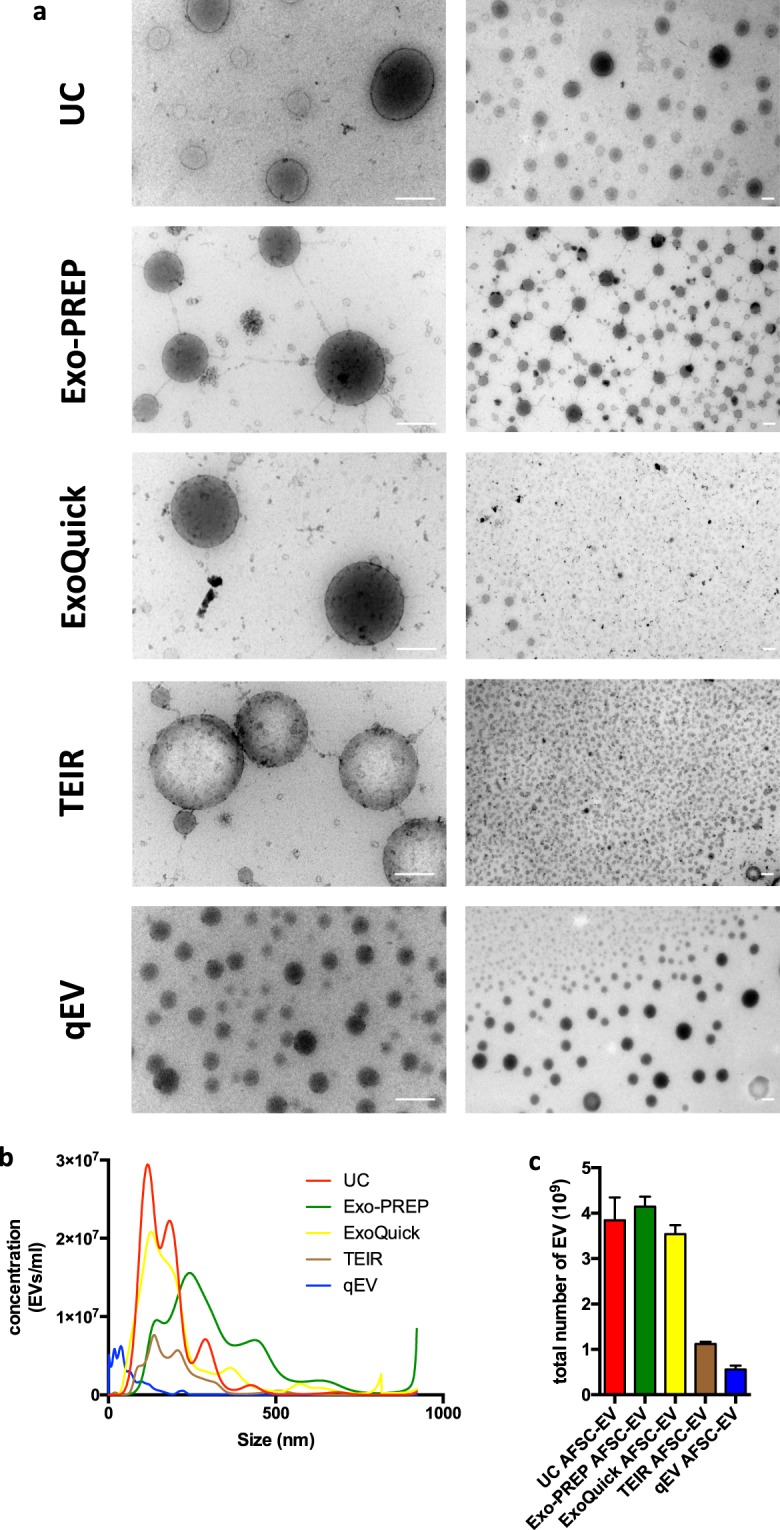
Comparison of EV morphology, size distribution, and yield with different isolation techniques. (**a**) Representative photos of AFSC-EV morphology analyzed by TEM; the two different magnifications highlight morphology of individual EVs at near fields (left column) and a population of EVs at far fields (right column) Scale bar = 200 nm. (UC = Ultracentrifugation; TEIR = Total Exosome Isolation Reagent). B: Representative plot of the average size distributions of AFSC-EVs isolated with the different techniques visualized using Nanoparticle Tracking Analysis. Data are representative of eight videos of AFSC-EV preparations. X-axis = size distribution (nm), y-axis = concentration (particles/ml). (**c**) Total particle yield calculated as the area under the curve from (**b**) of each AFSC-EV preparation.

AFSC-EVs isolated using the different techniques showed round-shape and double membrane morphology when examined with transmission electron microscopy (TEM; Fig. 1a). UC and qEV produced EV preparations that were almost free from background material, which instead was found in the reagent-based preparations (TEIR, Exo-PREP, ExoQuick).

#### Size

AFSC-EV size distributions determined by nanoparticle tracking analysis (NTA) varied with different isolation techniques. AFSC-EVs isolated with UC, TEIR, and Exo-Quick had similar size distributions (Fig. 1b). The smallest vesicles were isolated using size exclusion chromatography (qEV columns), which had the highest concentration of EVs <150 nm in size. Moreover, qEV isolation had fewer EVs >250 nm, indicating that this isolation technique could isolate the narrowest range of EV size. AFSC-EVs isolated with Exo-PREP had the largest size compared to the AFSC-EVs isolated with the other techniques.

#### Yield

UC, Exo-PREP, and ExoQuick generated the highest number of AFSC-EVs, whereas TEIR and qEV isolated the lowest concentrations of AFSC-EVs (Fig. 1c, Supplementary Table ST1).

### Different isolation techniques generated EV preparations with variable protein content, relative purities, and expression of EV markers

#### Total protein

**Figure 2:**
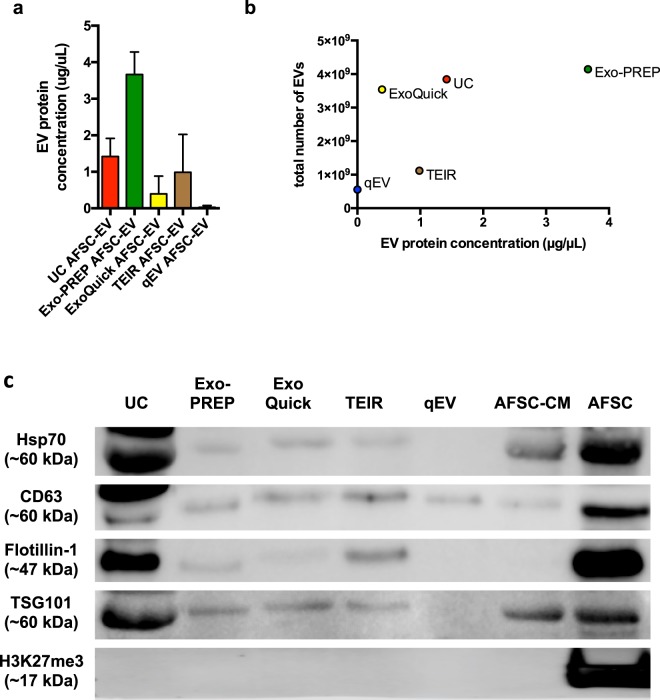
Comparison of AFSC-EV protein content and EV markers. (**a**) Protein quantification of AFSC-EV preparations using the Pierce Bradford assay. Data are shown as mean ± SD n = 3. No difference was found between preparations isolated with UC, ExoQuick and TEIR. AFSC-EV preparations isolated using qEV had lower protein content than those isolated using Exo-PREP (p < 0.05). (**b**) Correlation analysis between total number of particles analyzed with Nanoparticle Tracking Analysis (Fig. 1c) and EV protein concentration (μg/μL) in each preparation of AFSC-EVs obtained by different isolation techniques [p = 0.25, r = 0.6 (95% CI −0.56 to 0.97)]. (**c**) Expression of canonical EV markers Hsp70, CD63, Flotillin-1, and TSG101 obtained by Western blot analysis for the different isolation techniques. All AFSC-EV isolation techniques showed no evidence of residual cellular debris, as evidenced by a lack of H3K27me3 protein expression. AFSCs (parent cells) and AFSC-conditioned medium (AFSC-CM; the initial starting material from which all techniques were derived), are shown as positive controls. Representative photo from n = 3 replicate analyses.

The protein content was variable across the different AFSC-EV preparations (Fig. 2a). We found a similar protein content among the EV preparations obtained with UC, ExoQuick, and TEIR (UC vs. ExoQuick, p = 0.1; UC vs. TEIR, p = 0.5; ExoQuick vs. TEIR, p = 0.2), whereas, the EV protein content was significantly different between Exo-PREP and qEV isolations (p < 0.05).

#### Preparation purity

To define the purity of each preparation, we measured the ratio between the number of EVs obtained with each technique and the corresponding protein content. We did not observe a correlation between the total number of EVs isolated with each technique and the corresponding total protein content [p = 0.25, r = 0.6 (95% CI −0.56 to 0.97); Fig. 2b].

#### Expression of EV-related markers

AFSC-EV populations isolated with UC, Exo-PREP, ExoQuick, and TEIR had detectable levels of typical EV protein markers Hsp70, CD63, Flotillin-1, and TSG101 analyzed by Western Blot (Fig. 2c). qEV preparations only had detectable levels of CD63 protein expression. All AFSC-EV isolation techniques showed no evidence of residual cellular debris, as evidenced by a lack of H3K27me3 protein expression.

### AFSC-EV capability of regenerating injured lung epithelium

#### Matched doses of AFSC-EVs isolated with different techniques have different effects on lung epithelial cell survival

**Figure 3:**
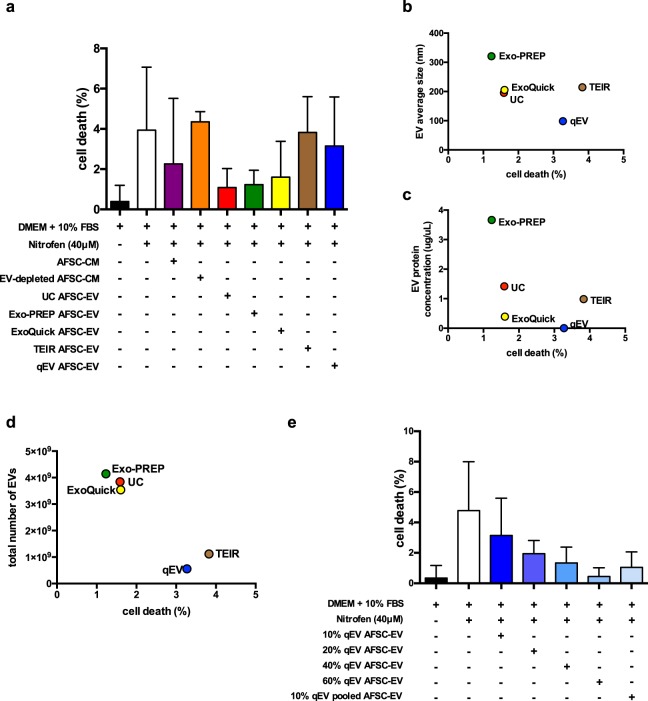
Regenerative capacity of AFSC-EVs isolated using different techniques in an *in vitro* model of lung injury. (**a**) Death rate of A549 cells in different conditions (Y axis). Compared to control (black bar), the rate of cell death increased with the administration of nitrofen (nitrofen group, white bar, p < 0.0001). The rate of cell death was brought back to normal levels by the administration of AFSC-conditioned medium (AFSC-CM, purple bar; p = 0.01 vs. nitrofen group; p = n.s. vs. control), ultracentrifuged AFSC-EVs (UC, red bar; p < 0.05 vs. nitrofen group; p = n.s. vs. control), ExoQuick AFSC-EVs (yellow bar; p < 0.01 vs. nitrofen group; p = n.s. vs. control), Exo-PREP AFSC-EVs (green bar; p < 0.001 vs. nitrofen group; p = n.s. vs. control). Conversely, the rate of cell death was not rescued using the supernatant of the ultracentrifuged CM (EV-depleted AFSC-CM, orange bar; p < 0.01 vs. control; p = n.s. vs. nitrofen group), Total Exosome Isolation Reagent AFSC-EVs (TEIR, brown bar; p < 0.0001 vs. control; p = n.s. vs. nitrofen group), or qEV AFSC-EVs (blue bar; p < 0.0001 vs. control; p = n.s. vs. nitrofen group). Conditions of each treatment, conducted in at least n = 4 experiments, are shown below the x-axis. (**b**) No correlation was found between EV size and the rate of cell death [p = 0.3, r = −0.6 (95% CI −0.97 to 0.6)]. (**c**) No correlation was found between EV protein concentration and the rate of cell death [p = 0.3, r = −0.6 (95% CI −0.96 to 0.6)]. (**d**) There was a negative correlation between total number of EV particles and the rate of cell death [p = 0.01, r = −0.97 (95% CI −0.99 to −0.48)]. (**e**) Dose curve analysis of AFSC-EVs isolated with qEV and administered to nitrofen-injured A549 cells. The rate of cell death decreased proportionally by increasing the concentration of AFSC-EVs. Treatment with > 40% by volume AFSC-EVs brought the rate of cell death back to control levels (p = n.s. vs. control). In addition, using 10% by volume qEV from pooled fractions 7 to 11 also reduced rate of cell death back to control levels (p = n.s. vs. control). Data are shown as mean ± standard deviation and are representative of the following number of biological replicates per condition: Control, n = 16; nitrofen, n = 14; 10% UC AFSC-EVs, n = 14; 10% Exo-PREP AFSC-EVs, n = 5; 10% ExoQuick AFSC-EVs, n = 4; 10% TEIR AFSC-EVs, n = 5; 10% AFSC-CM, n = 3; 10% EV-depleted AFSC-CM, n = 3; 10% qEV AFSC-EVs, n = 9; 20% qEV AFSC-EVs, n = 3; 40% qEV AFSC-EVs, n = 3; 60% qEV AFSC-EVs, n = 3; 10% qEV AFSC-EVs pooled fraction 7–11, n = 5.

To evaluate if different EV isolation techniques could have an effect on AFSC-EV mediated regenerative potential, we tested them as treatment on a previously described *in vitro* epithelial cell model of lung injury^29^. In this model, cell death is induced in alveolar epithelial type 2 cells via the administration of nitrofen^29^. We confirmed that nitrofen administration to A549 cells significantly increased the rate of cell death (DMEM only = 0.4 ± 0.8%, nitrofen = 4 ± 3%; p < 0.0001; Fig. 3a). The administration of AFSC-CM (cell free-, EV-containing supernatant) to nitrofen-injured A549 cells significantly reduced the rate of cell death back to control levels (AFSC-CM = 2.3 ± 3%; AFSC-CM vs. nitrofen, p < 0.01; p = n.s vs. DMEM only). When AFSC-CM was depleted of EVs (supernatant from ultracentrifugation), a reduction in the rate of cell death was no longer observed (4.4 ± 0.5%; p = n.s. vs. nitrofen). The rate of cell death of nitrofen-injured A549 cells treated with AFSC-EVs isolated with UC (1.3 ± 0.9%), ExoQuick (1.6 ± 1.7%) and Exo-PREP (1.2 ± 0.7%) was lower than that of untreated nitrofen-injured A549 cells (p < 0.0001 for UC and Exo-PREP; p = 0.002 for ExoQuick) and not different from that of control cells (p = n.s.; Fig. 3a). Conversely, TEIR and qEV isolated AFSC-EVs did not reduce the rate of cell death of nitrofen-injured A549 cells (TEIR: 3.8 ± 1.8%; qEV: 3.1 ± 2.4%; p = n.s. to nitrofen).

#### The effect of AFSC-EVs on lung epithelial cell survival depends on the number of EVs administered

We investigated whether variations in lung epithelial survival were due to EV characteristics that differed among the AFSC-EV preparations, i.e. EV size, protein content, and number of EVs. The rate of cell death did not correlate with the size [p = 0.3, r = −0.6 (95% CI −0.97 to 0.6); Fig. 3b] or the protein content [p = 0.3, r = −0.6 (95% CI −0.96 to 0.6); Fig. 3c] of AFSC-EVs isolated from each method. Conversely, the EV count of AFSC-EVs isolated with all techniques negatively correlated with the rate of cell death [p = 0.01, r = −0.97 (95% CI −0.99 to −0.48); Fig. 3d].

#### AFSC-EV capability of regenerating injured lung epithelium is preserved by increasing the dose of administered EVs

To validate that the number of EVs is important for AFSC-EV biological effect, we investigated if increasing the dosage of the AFSC-EV preparation with the lowest concentration (qEV) could reduce the rate of cell death. We found that treatment with AFSC-EVs isolated by qEV reversed the rate of cell death back to control levels with >40% by volume (1.3 ± 1%, p = n.s. vs. control; Fig. 3e]. Moreover, when multiple fractions of qEV preparations were pooled (7–11), the cell death rate was significantly reduced to control levels (p = n.s. to control, p < 0.05 to nitrofen). We also investigated whether the expression of an EV related marker would correlate to the number of EV particles and to the cell death rate, and we did not find a significant correlation (Supplementary Fig. S1A,B). Lastly, we investigated if decreasing volumes of AFSC-EVs had the opposite effect observed at 10% by volume treatment by administering UC AFSC-EVs in decreasing doses from 10% to 1.25%. We observed that AFSC-EVs from 5%, 2.5%, and 1.25% by volume were able to rescue cell death back to control levels, though the rate of cell death showed an increasing trend that was not significantly different (Supplementary Fig. S2A).

To confirm the impact of the isolation strategies on EV biological properties, we also compared the effect of the different preparations on cell migration with a scratch assay. Nitrofen injury significantly impaired the ability of A549 cells to migrate by approximately 55% (p = 0.002 compared to control; Supplementary Fig. S2B). Administration of AFSC-EVs from all preparations improved cell migration rate back to control levels (p = n.s. relative to control; p = 0.008 nitrofen vs. UC AFSC-EVs; p = 0.07 nitrofen vs. Exo-PREP AFSC-EVs; p = 0.04 nitrofen vs. ExoQuick AFSC-EVs; p = 0.02 nitrofen vs. TEIR AFSC-EVs; p = 0.04 nitrofen vs. qEV AFSC-EVs) with a beneficial impact that had a similar trend as observed in cell death rate.

## Discussion

The present study shows that commonly used EV isolation techniques can isolate AFSC-EVs that have typical EV morphology and protein markers. However, AFSC-EV size, protein content, preparation purity, and number of EVs varied between different isolation techniques. We also noticed that AFSC-EVs isolated with different techniques had different effects on a model of damaged lung epithelium. Interestingly, among all the EV characteristics, the number of EVs administered was the most important parameter responsible for AFSC-EV regenerative potential.

The EV isolation techniques employed in this study are commonly used methods in the field. The variability observed with these techniques have also been reported by other authors evaluating isolation methods for EVs from other sources beyond AFSCs^30,31^. Although our experimental methods did not include an analytical technique to separate AFSC-EVs from conditioned medium based on EV size distributions, we found that the variable EV size distribution did not have a significant impact on AFSC-EV regenerative potential in an *in vitro* lung injury model. EVs represent a heterogeneous mixture of vesicles of different sizes released from prokaryotic and eukaryotic cells^32^. It is not fully understood how size contributes to biological function of EVs. Among all EVs, exosomes (~30 to 100 nm in size)^20,33^ have been classically considered the subpopulation with potent, protective, and pathological functions, and therefore with higher potential as diagnostic or therapeutic tools^34^. For instance, Keerthikumar and colleagues demonstrated a stronger proliferative and pro-migratory effect of exosomes than larger EVs secreted by neuroblastoma cell lines^35^. However, some studies have shown that EVs of other sizes can be more effective than exosomes in a given biological context. For example, Minciacchi *et al*. reported that large EVs (1–10μm) were more efficient than small EVs secreted by prostate cancer cell lines in fibroblast reprogramming and endothelial cell tube formation^36^. In order to better address the significance of EV-sizes being able to impact their biological effect, focus has now shifted to developing technologies that isolate EV-preparations with smaller size distributions or that determine the relative proportion of EVs of different sizes more accurately in a given sample^34^.

In our study, size did not appear to correlate with AFSC-EV regenerative capacity as preparations with a similar mean EV size (ExoQuick, UC, and TEIR) had different biological effects in the lung injury model. Further suggestion that size is not essential for AFSC-EV potential comes from the dose curve experiment (Fig. 3e). When we treated injured lung epithelium with the smallest and least abundant EV preparation (qEV), we found little to no protection. However, when we increased the number of AFSC-EVs from the same preparation and matched the concentration of larger EV preparations (UC, ExoQuick, Exo-PREP), we observed a similar protective effect.

Additional evidence that specifically AFSC-EV-size does not dictate biological function can be found in the literature, where different isolation techniques led to collection of AFSC-EVs of variable sizes, but all equally effective in various experimental models (Table 2)^6,22–26^. Using UC, Romani *et al*. isolated human AFSC-EVs of 40–200 nm, Mellows *et al*. reported human AFSC-EVs with an average size of 73 nm, Sedrakyan *et al*. isolated a heterogeneous population of mouse AFSC-EVs ranging from 100 nm to 400 nm, and Balbi *et al*. reported human AFSC-EVs ranging from 100–600 nm. Moreover, Xiao *et al*. described mouse AFSC-EVs with an average size of 66 nm isolated with ExoQuick. As such it appears that EVs of different sizes may deliver beneficial regenerative effects.

**Table 2:** Studies reporting the isolation, characterization, and use of Amniotic Fluid Stem Cell-Extracellular Vesicles (AFSC-EVs).

Reference	Species	AFSC isolation	EV isolation technique	Model	Biological effect
Romani *et al*.^22^	Human	Morphologic selection and confirmation with FACS and RT-PCR	UC	*In vitro:* Immuno-modulation of human peripheral blood mononuclear cells	Decrease in T-cell proliferation and expression of immune-modulatory protein IDO1
Xiao *et al*.^23^	Mouse	FACS of AFSCs expanded in culture	ExoQuick-TC	*In vitro:* nitrogen-mustard injured mouse granulosa cells	Decrease in granulosa cell apoptosis via microRNA10a
				*In vivo:* chemotherapy-induced ovarian failure in mice	Decrease in granulosa cell apoptosis and preservation of ovarian follicles
Balbi *et al*.^24^	Human	c-Kit+ immunoselection (MACS)	UC	*In vitro:* C2C12 mouse myoblast cells, human dermal fibroblasts, human peripheral blood mononuclear cells	Inhibition of C2C12 apoptosis, increase in fibroblast proliferation and immunomodulation of peripheral blood mononuclear cells
				*In vivo:* skeletal muscle atrophy in mice	Decrease in skeletal muscle inflammation
Mellows *et al*.^25^	Human	c-Kit+ immunoselection (MACS)	UC	*In vitro:* human glioblastoma (U251) cells	Decreased inflammation by suppression of NF-kB signaling
				*In vivo:* acute muscle injury model in mice	Angiogenesis and muscle fiber regeneration
Sedrakyan *et al*.^6^	Mouse	c-Kit+ immunoselection (MACS)	UC (+FACS)	*In vitro:* mouse glomerular endothelial cells	Protection against VEGF-induced glomerular endothelial cell damage
				*In vivo:* mouse model of Alport syndrome	Improvement of renal physiological parameters (proteinuria and serum creatinine)
Beretti *et al*.^26^	Human	c-Kit+ immunoselection (MACS)	TEIR	*In vitro:* Immuno-modulation of human peripheral blood mononuclear cells	Decrease in T-cell proliferation
This study	Rat	c-Kit+ immunoselection (MACS)	UC, Exo-PREP, ExoQuick, TEIR, qEV	*In vitro*: human alveolar epithelial type 2 (A549) cells stressed with nitrofen	Decrease in A549 cell apoptosis

FACS: Fluorescence Activated Cell Sorting.

RT-PCR: Reverse transcription polymerase chain reaction.

UC: Ultracentrifugation.

IDO1: Indoleamine 2,3-dioxygenase.

MACS: Magnetic Activated Cell Sorting.

VEGF: Vascular endothelial growth factor.

TEIR: Total Exosome Isolation Reagent.

Having established that EV size distribution was not crucial to AFSC-EV mediated biological responses, we next assessed the role of EV protein content. In our study, we found variability in the total protein content of AFSC-EVs, with a significant difference between the preparation with the highest protein content (Exo-PREP) and the one with the lowest (qEV) (Fig. 2b). The ratio between protein content and the number of EVs has been previously used as an indicator of EV purity^37^. In our study, ExoQuick had the lowest protein concentration with highest number of particles isolated in keeping with a high degree of EV-purity. Conversely, qEV was the preparation with the lowest protein content and the least number of particles, which might be explained by obligatory dilution of EV containing samples by SEC.

As also observed for EV size, protein content does not appear to influence the regenerative capability of AFSC-EVs. In fact, the dose curve experiment (Fig. 3e) showed that higher doses of qEV isolated AFSC-EVs had similar biological effects on a lung injury model as preparations with higher protein content.

Lastly, we found variability in the total number of AFSC-EVs isolated using different techniques. This variability has also been reported in other studies, where different techniques were assessed to isolate other EV populations^31,38^. In particular in our study, the highest EV-yields were obtained with UC, ExoQuick and Exo-PREP. Interestingly, when we tested the effects of equal volumetric additions of AFSC-EVs isolated with different techniques on the *in vitro* model of lung injury, we noticed that only AFSC-EV preparations with a high particle yield (UC, ExoQuick and Exo-PREP) had a beneficial effect in reducing cell death. This finding suggested a possible dose effect related to the number of EVs isolated, as also supported by the negative correlation of EVs isolated with the cell death rate. To test dose responsiveness, we elected to titrate increasing amounts of the smallest- least protein containing-EVs (qEV) into the nitrofen lung injury model. We aimed to deliver doses of the qEV isolated EVs to obtain numbers of EVs which would resemble the other isolation techniques (Supplementary Table ST2). By adding 20% by volume of the qEV group, we approximated the TEIR group, while adding 60% by volume more closely resembled the UC, ExoQuick and Exo-PREP groups (Supplementary Table ST2). Increasing the number of qEV derived EVs increased the cell survival advantage in the lung injury model in a dose dependent fashion. We confirmed this response by pooling additional fractions eluted by qEV columns included more EVs, and obtaining a reduction on cell death rates similar to >40% treatment by volume. This confirmed that EV count is crucial for the biological effect of an EV preparation, regardless of the isolation technique used. Moreover, we elected to titrate decreasing amounts of UC AFSC-EV preparations into the nitrofen lung injury model, as this was one of the preparations that brought the rate of cell death back to control levels. Interestingly, when AFSC-EVs isolated using UC were administered in decreasing doses, we observed a trend towards an increase in cell death rate, albeit not significantly different.

Lastly, we investigated the effects of different AFSC-EV preparations on another outcome measure, such as cell migration. We specifically studied cell migration as an outcome measure of tissue homeostasis, as nitrofen exposure impairs lung epithelial cell migration^39^, and EV administration has been reported to be beneficial in cell culture^40^. Interestingly, we observed a positive effect on the ability of nitrofen-injured A549 cells to migrate with the addition of all AFSC-EV preparations to varying degrees (Supplementary Fig. 2B).

The concept that EVs have variable dose-dependent effects has also been observed in other disease models^41–43^. In a model of central nervous system injury, Tassew *et al*. demonstrated that retinal neurons respond to EVs in a dose-dependent manner *in vitro* and *in vivo*^41^. Likewise, Tabak *et al*. showed that in an *in vitro* model used to study the human ocular drainage system, exposure to different concentrations of EVs derived from non-pigmented ciliary epithelium resulted in a dose–dependent effect on the Wnt signaling^42^. Recently, in an *in vitro* model of lung injury similar to the one herein tested, Willis *et al*. also reported that mesenchymal stem cell derived EVs dose dependently regulated TNFα expression in alveolar macrophages^43^. The concept of EV dose-dependent effect has lately become more topical, as we are approaching an era of EV based therapeutics, that involves the consideration of how EV storage and stability affect EV biological functions^44^. In an effort to reduce variability in EV preparations, some authors have proposed to develop tools to estimate the efficacious EV dose, including fingerprinting assays and potency assay^45^. EV dose could be quantified by fingerprinting assays using surrogate indicators, such as EV markers or microRNAs, and/or by potency assays assessing the ability of a preparation to elicit the desired biologic effect *in vitro* or *in vivo*^45^. Although standardization of EV isolation is unlikely to be achieved due to variables in study design and starting material, there is great value in reporting detailed descriptions during comparative studies as described by the broader EV community^46–48^.

The present study confirms that AFSC-EVs offer potential as beneficial effectors in regenerative medicine. In recent years, EVs have gained significant interest in the field of regenerative medicine, as they exert an effect that is similar and sometimes greater than that of their parent cells^49,50^. To date, the effects of AFSC-EVs have been investigated mainly in models with translational potential towards clinical application, such as chemotherapy-induced ovarian failure^23^, skeletal muscle atrophy^24^, and Alport syndrome^6^. In these studies, AFSC-EVs have shown immunomodulatory^22,26^, proangiogenic^6,25^, antiapoptotic^23,24^ and anti-inflammatory effects^24,25^. In the present study, we have used an *in vitro* model of lung injury as a platform to test different EV isolation techniques. Interestingly, we have also observed for the first time that AFSC-EVs have a beneficial anti-apoptotic effect on injured respiratory epithelium. It has been reported that AFSCs hold regenerative potential in the lung, as they can integrate and differentiate into epithelial lung lineages^9^, reduce lung fibrosis^51^, and repair damaged alveolar epithelial type 2 cells^52^, which are important for surfactant production^53^. Moreover, AFSCs have a reparative effect in nitrofen-mediated models of pulmonary hypoplasia via an undetermined paracrine mechanism^10,54^. In the present study, using an established nitrofen-mediated model of lung injury, we have shown for the first time that AFSC-EVs are the paracrine effector of AFSCs in lung disease. This was confirmed by the lack of beneficial effect observed when EV-depleted AFSC-CM was used.

In conclusion, techniques that are based on differential sedimentation, solubility or size-exclusion chromatography are able to isolate AFSC-EVs with typical EV morphology and protein markers. The variability observed in EV size and protein content did not significantly affect AFSC-EV biological function. Conversely, EV count influenced AFSC-EV beneficial effect on a model of lung injury, in a dose dependent fashion. We advise other investigators working with EVs to consider EV concentration as a variable that could have an impact on AFSC regenerative potential.

Moreover, in the present study, we have shown for the first time that AFSC-EVs exert a beneficial effect in an *in vitro* model of lung injury. This adds to the current literature, where AFSC-EVs have been reported to hold great potential in a variety of disease models. Further studies are needed to understand the beneficial effect that AFSC-EVs may exert in the context of lung disease.

## Materials and Methods

### Isolation of AFSC-EVs from cell culture

#### AFSC cell culture

Amniotic fluid stem cells (AFSCs) were isolated from a pregnant rat at embryonic day E12 by c-Kit+ selection as previously described^2^. In brief, AFSCs were grown to 90% confluence in a humidified 37 °C, 5% CO_2_ chamber in alpha-minimal Essential Media (αMEM, Gibco, ThermoFisher, Waltham, MA) supplemented with 20% Chang supplements (Irvine Scientific, Santa Ana, CA), 15% fetal bovine serum (FBS, ThermoFisher Scientific, Waltham, MA), and 0.5% Penicillin/Streptomycin (ThermoFisher Scientific, Waltham, MA). AFSCs were cultured for 18 hours in 7.5% exosome-depleted FBS (ThermoFisher, Waltham, MA) in αMEM. For each of the EV isolation methods described below, 2 mL of AFSC-CM from 4 × 10^6^ AFSCs was used. To eliminate the possibility that bacterial contaminants contributed to EVs in the conditioned medium, we confirmed that AFSCs were mycoplasma free (PCR Mycoplasma Detection Kit, Richmond, BC).

#### Ultracentrifugation isolation of AFSC-EVs

Residual cells and debris were cleared from AFSC-CM by differential centrifugation at 300 × g followed by 1200 × g both for 10 minutes at room temperature. Next, the supernatant was filtered (0.20 µm cellulose filter, Corning, NY) then ultracentrifuged at 100,000 × g for 14 h at 4 °C (swing bucket rotor on minimum acceleration/break setting, SW 32Ti Beckman Coulter, Brea, CA). Post-ultracentrifugation the pellet was resuspended in 500 μL of phosphate buffered saline and either used immediately or stored at −20 °C for up to six months. Supernatants obtained from the ultracentrifugation was used as EV-depleted CM.

#### Reagent-based isolation of AFSC-EVs

TEIR (ThermoFisher Scientific, Waltham, MA), ExoQuick-TC (System Biosciences, Palo Alto, CA) and Exo-PREP (HansaBioMed, Basel, Switzerland) kits were used as per the manufacturers’ recommended protocols. The AFSC-EVs preparations were re-suspended in 500 μL of phosphate buffered saline and either used immediately or stored at −20 °C for up to six months.

#### Size-exclusion chromatography isolation of AFSC-EVs

Size-exclusion chromatography (SEC) was used to isolate EVs from pre-cleared AFSC-CM using qEV (IZON, Cambridge, MA) as per manufacturer’s protocol. In all experiments, Fraction 7 was collected and used for subsequent EV characterizations and experiments as per manufacturer’s recommendations. The AFSC-EVs preparations were either used immediately or stored at −20 °C for up to six months.

#### Characterization of AFSC-EVs isolated by different methods

AFSC-EVs obtained using the different isolation strategies (Table 1) described above were characterized for morphology, size, protein concentration, and expression of EV markers.

#### TEM

AFSC-EV morphology was assessed using TEM. For TEM EV-analyses, AFSC-EV preparations from each isolation strategy were mixed with equal volumes of 4% paraformaldehyde in PBS (Electron Microscopy Sciences, Hatfield, PA), and adhered to formvar-carbon coated copper grids described below. These grids were formed with 0.5% formvar solution (Electron Microscopy Sciences, Hatfield, PA) from powder in ethylene dichloride. A glass slide was dipped in the formvar, allowed to dry, its edges were scored then pushed into a water bath to float the film off the slide. The grids were then placed on the film, and flipped out of the water with parafilm. The sheet of formvar coated grids were then placed in a Cressington carbon evaporator where carbon was applied. EV preparations were fixed again with 2% glutaraldehyde in PBS, contrasted with uranyl-oxalate (Electron Microscopy Sciences, Hatfield, PA) and embedded in methyl cellulose-UA. AFSC-EVs were visualized on a Tecnai 20 (FEI, Hillsboro, OR) from 25 kx to 100 kx magnification.

#### Nanoparticle Tracking Analysis

To determine EV sizes, 300 µL of AFSC-EVs (undiluted EVs from the different isolation strategies) were analyzed by the NanoSight LM10 system (NanoSight Ltd, Salisbury, UK). 30-second videos of EVs (number of experiments n = 3, number of videos n = 8) were collected, averaged, and analyzed using LM10 NTA equipped with a 65 mWatt 405 nm violet laser (NanoSight Ltd, Salisbury, UK). For size calibration, 100 nm polystyrene beads (Malvern Instruments, Saint-Laurent, Canada) were used as previously described^55^. At capture with sCMOS camera on NTA 3.1 (machine) Build 3.1.46 (software), the temperature was 22 **°**C.

#### Protein analysis and relative purity of EV preparations

To investigate the total protein yield of the different AFSC-EV preparations, we used the Pierce Bradford assay (ThermoFisher Scientific, Waltham, MA). To estimate the relative purity of AFSC-EVs obtained from the different isolation strategies, the ratio of particle counts measured by NTA (number of EV particles/mL) divided by protein concentration (µg/µL) measured using the Pierce Bradford assay was compared, as previously described^37^.

#### Expression of EV protein markers

To further characterize the AFSC-EVs, we performed a Western blot analysis of canonical EV markers on AFSC-EVs isolated with the different methods as well as with AFSCs and AFSC-CM as positive controls. Hsp70, CD63, Flotillin-1, and TSG101 were measured from AFSC-EVs, and from 20 µg total protein from AFSC-CM, and AFSCs using ExoAb Antibody Kit for Hsp70 (System Biosciences, Palo Alto, CA; primary antibody: rabbit anti-human, 1:1,000 dilution; secondary antibody: goat anti-rabbit HRP, 1:10,000 dilution), CD63 (System Biosciences, Palo Alto, CA; primary antibody: rabbit anti-human, 1:1,000 dilution; secondary antibody: goat anti-rabbit HRP, 1:10,000 dilution), TSG101 (Santa Cruz Biotechnology, Dallas, TX; primary antibody mouse anti-rat, 1:500 dilution; secondary antibody: goat anti-mouse HRP, 1:3,000 dilution), and Flotillin-1 (BD Transduction Laboratories, San Jose, CA; primary antibody mouse anti-rat, 1:1,000 dilution; secondary antibody: goat anti-mouse HRP, 1:3,000 dilution). To ensure that the EV preparations did not include nuclear proteins (indicative of cellular-contamination), we assessed anti-H3K27me3 (Diagenode, Denville, NJ; primary antibody rabbit anti-rat, 1:200 dilution; secondary antibody: goat anti-rabbit HRP, 1:1,000 dilution) expression as a negative control. 40uL of each preparation was reduced with 4X reducing agent and 10X sample buffer, and loaded onto SDS-PAGE gels. PageRuler™ plus prestained protein ladder (ThermoFisher Scientific, Waltham, MA) was used. Proteins were transferred to polyvinylidene difluoride membranes, which were pre-blocked in 5% nonfat milk in tris-buffered saline and Tween (TBST, Sigma Aldrich, St. Louis, MO), washed in TBST, and incubated with primary and secondary antibodies. Blots were visualized using enhanced chemo-luminescence (Pierce™, ECL Western Blotting Substrate, ThermoFisher Scientific, Waltham, MA).

#### Regenerative potential of AFSC-EVs in a model of lung injury

Immortalized adenocarcinomic alveolar basal epithelial cells (A549; Sigma Aldrich, St. Louis, MO) were grown until confluence in Dulbecco Modified Eagle Medium F-12 (DMEM, ThermoFisher Scientific, Waltham, MA) supplemented with 10% FBS and 0.5% Penicillin/Streptomycin (ThermoFisher Scientific, Waltham, MA). A549 cells were injured with nitrofen (40 μM in DMSO; Sigma Aldrich, St. Louis, MO) for 18 h. After this time, select groups of injured A549 cells were then treated with 10% by volume of AFSC-EVs in DMEM from the different isolation methods. Control groups included: uninjured and untreated (no nitrofen, no AFSC-EVs, DMEM only) A549 cells and injured but untreated (nitrofen only, no AFSC-EVs) A549 cells. After 24 hours, cell death rates were assessed in all groups. Live A549 cells were identified by calcein staining (1 μM) while dead cells were identified with ethidium staining (3 μM) (Live/Dead^TM^ cytotoxicity kit, ThermoFisher Scientific, Waltham, MA). Five 20X magnification fields of cells were imaged using an inverted fluorescence microscope (Leica DMI6000B, Wetzlar, Germany) per replicate were assessed by two independent researchers, and averaged to identify the percentage of dead cells.

To assess whether different isolation techniques had an effect on the AFSC-EV regenerative potential based on EV characteristics such as size and yield, we correlated these two with the rate of cell death.

Finally, we performed a dose analysis of the AFSC-EV isolated using qEV by titrating 10%, 20%, 40%, and 60% of the preparation by volume in DMEM as treatment to nitrofen-injured A549 cells. We also administered decreasing doses of AFSC-EVs isolated using UC at 10%, 5%, 2.5%, and 1.25% by volume treatment. Live/Dead^TM^ cytotoxicity assay was performed as described above after 24 h treatment with increasing doses of AFSC-EV isolated with qEV and decreasing doses of AFSC-EV isolated with UC.

To test the ability of AFSC-EV in promoting cell migration, an artificial would was created in cell culture wells on nitrofen-injured cells with a sterile pipette tip. Nitrofen-injured cells were then treated with AFSC-EVs isolated from the different methods at 10% by volume. Control cells did not receive the nitrofen injury prior to the formation of an artificial wound. Cells were incubated with Hoechst 33342 (1:2000 in PBS; ThermoFisher, Waltham, MA) for 10 minutes at room temperature. Immunofluorescence imaging was conducted after 6 hours and 12 hours to assess the rate of cell migration in each experimental group in two biological replicates. At least n = 25 fields at the cell front were taken per condition.

#### Statistics

Comparisons between groups were conducted using Kruskal-Wallis one-way analysis of variance with Dunn’s multiple comparison test. Data are reported as mean ± standard deviation. For correlation analysis, we calculated the Pearson correlation coefficient and reported the p-value, Pearson r, and 95% confidence interval. All experiments were performed at least three times. All statistical tests were performed using GraphPad PRISM Version 6.0e.

## Supplementary information

Supplementary Info

## Data Availability

All data generated or analyzed during this study are included in this published article. We have submitted relevant data of our experiments to the EV-TRACK knowledgebase (EV-TRACK ID: EV180061).
