# Supplementary material for: The Regenerative Potential of Amniotic Fluid Stem Cell Extracellular Vesicles: Lessons Learned by Comparing Different Isolation Techniques
Source: Sci Rep. 2019 Feb 12;9:1837. doi: 10.1038/s41598-018-38320-w (PMC6372651; doi:10.1038/s41598-018-38320-w)
Supplement: Supplementary file 1 — Supplementary Info [file 41598_2018_38320_MOESM1_ESM.docx]

**The Regenerative Potential of Amniotic Fluid Stem Cell Extracellular Vesicles: Lessons Learned by Comparing Different Isolation Techniques**

Lina Antounians^1,2^, Areti Tzanetakis^1,2^, Ornella Pellerito^1,2^, Vincenzo D Catania^1,2^, Adrienne Sulistyo^1,2^, Louise Montalva^1,2^, Mark J. McVey^3^, Augusto Zani^1,2*^

^1^ Developmental and Stem Cell Biology Program, Peter Gilgan Centre for Research and Learning, The Hospital for Sick Children, Toronto, M5G 0A4, Canada

^2^ Division of General and Thoracic Surgery, The Hospital for Sick Children, Toronto, M5G 1X8, Canada

^3^ Department of Anesthesia and Pain Medicine, The Hospital for Sick Children, Department of Anesthesia, University of Toronto, Toronto, M5G 1X8, Canada.

*** Corresponding author**

Augusto Zani, MD, PhD

Division of General and Thoracic Surgery

The Hospital for Sick Children

1524C-555 University Ave

Toronto, ON M5G 1X8, Canada

Phone +1-416-813-7564 - ext. 202413

Fax +1-416-813-7477

E-mail: [augusto.zani@sickkids.ca](mailto:augusto.zani@sickkids.ca)

**Supplementary Figure S1:** Correlation analysis of protein expression with total number of EVs and cell death rate. Intensity bands of CD63, Flotillin-1, TSG101, and Hsp70 expression with Western blot analyses were correlated to A) total number of EVs determined by nanoparticle tracking analysis from **Fig. 1C**, and B) cell death rates in the *in vitro* assay of lung injury **Fig.3A**. The protein content of EV markers CD63, Flotillin-1, TSG101, and Hsp70 did not have a significant correlation with either total number of EVs (CD63, p=0.78; Flotillin-1, p=0.53; TSG101, p=0.38; Hsp70, p=0.43), or cell death rate (CD63, p=0.94; Flotillin-1, p=0.63; TSG101, p=0.47; Hsp70, p=0.51).

**Supplementary Figure S2:** Additional effects of AFSC-EVs on cell death and cell migration. A) cell death rates with administration of decreasing doses of UC AFSC-EVs (10% to 1.25%) on nitrofen-injured A549 cells. AFSC-EVs from 5%, 2.5%, and 1.25% by volume were able to rescue cell death back to control levels, though the rate of cell death showed an increasing trend that was not statistically different. B) cell migration rate (μm/hour) of A549 cells injured with nitrofen and treated with the different AFSC-EV preparations. Administration of AFSC-EVs from all preparations improved cell migration rate back to control levels (p=n.s. relative to control; p=0.008 nitrofen vs. UC AFSC-EVs; p=0.07 nitrofen vs. Exo-PREP AFSC-EVs; p=0.04 nitrofen vs. ExoQuick AFSC-EVs; p=0.02 nitrofen vs. TEIR AFSC-EVs; p=0.04 nitrofen vs. qEV AFSC-EVs).

**Supplementary Figure S3:** Un-cropped images of Western blot analyses from Fig. 2C.

**Supplementary Table ST1. Concentrations of isolated AFSC-EVs determined by Nanoparticle tracking analysis.**

| **Method** | **Total EV count** | **Mean size (nm)** | **Mode size (nm)** |
| --- | --- | --- | --- |
| **UC** | 3.844×10^9^ | 181.8 ± 7.3 | 117 ± 5.1 |
| **ExoQuick** | 3.540×10^9^ | 217.5 ± 2.3 | 137.8 ± 10.8 |
| **TEIR** | 1.117×10^9^ | 205.5 ± 3.1 | 134.8 ± 15.3 |
| **Exo-PREP** | 4.145×10^9^ | 328.8 ± 6.6 | 467 ± 227.7 |
| **qEV** | 5.555×10^8^ | 53.0 ± 9.9 | 21.4 ± 20.4 |

UC: ultracentrifugation

TEIR: Total Exosome Isolation Reagent

CM: conditioned medium

PBS: phosphate buffered saline

**Supplementary Table ST2. Approximation of the number of EVs present in each preparation administered to nitrofen-injured A549 cells determined by Nanoparticle tracking analysis.**

| **AFSC-EV preparation** | **Percentage by volume** | **Approximate number of EVs** |
| --- | --- | --- |
| UC | 10% | 192,200,000 |
| ExoQuick | 10% | 177,000,000 |
| TEIR | 10% | 55,850,000 |
| Exo-PREP | 10% | 207,250,000 |
| qEV | 10% | 27,775,000 |
| qEV | 20% | 55,550,000 |
| qEV | 40% | 111,100,000 |
| qEV | 60% | 166,650,000 |

UC: ultracentrifugation

TEIR: Total Exosome Isolation Reagent
